# Determinants of malaria vaccine willingness among caregivers of children under five years in Akoko, Southwestern Nigeria

**DOI:** 10.5281/zenodo.22045850

**Published:** 2026-08-21

**Authors:** Oluwaremilekun G. Ajakaye

**Affiliations:** 1 Department of Public Health, Adekunle Ajasin University, Akungba Akoko, Ondo State, Nigeria.; 2 Laboratory of Molecular Parasitology and Genomics of Neglected Tropical Diseases, Adekunle Ajasin University, Akungba Akoko, Ondo State, Nigeria.

## Abstract

**Background:**

Malaria remains a significant public health challenge in Nigeria, especially among children under five years. The recent introduction of WHO-recommended malaria vaccines offers renewed hope for reducing malaria-related morbidity and mortality, but caregivers' willingness to vaccinate their children depends on their awareness, perceptions and practices. Guided by the Health Belief Model (HBM), this study investigated caregivers' willingness to vaccinate their children against malaria and the factors associated with this willingness in the Akoko area of Ondo State, Nigeria.

**Materials and Methods:**

A community-based cross-sectional study was conducted among 498 caregivers of children aged 0-59 months across eight rural and semi-urban communities in Akoko, selected using a multi-stage sampling technique. Data were collected using a structured, pre-tested questionnaire alongside malaria testing using rapid diagnostic test (RDT) kits, and analysed with Microsoft Excel and R software using descriptive and inferential statistics, including multivariable logistic regression.

**Results:**

The mean age (± SD) of caregivers was 34.5±11.0 years, and most were female (87.1%). Most caregivers reported appropriate malaria preventive practices, particularly mosquito net ownership, use of insecticide-treated nets, and environmental sanitation; preventive practice was significantly associated with caregiver education (p < 0.05). Malaria prevalence among under-five children was 10.1% and varied significantly with age (p = 0.008). Awareness of the malaria vaccine was high (77.9%), predominantly sourced from the health system, and willingness to vaccinate was high (87.9%). The mean overall HBM score was 48.3±13.9 (out of 60). Willingness to vaccinate was significantly associated with religion, trust in vaccine information and HBM category (p = 0.014, p = 0.002, p < 0.001, respectively). In multivariable analysis, caregivers who did not trust the information they received (AOR = 0.16; 95% CI: 0.05-0.53; p = 0.002) and those whose previous vaccination experiences influenced their decision (AOR = 0.24; 95% CI: 0.09-0.71; p = 0.009) had lower odds of willingness to vaccinate, while a higher HBM score was associated with higher odds of willingness (AOR = 1.04; 95% CI: 1.00-1.08; p = 0.030).

**Conclusions:**

Malaria preventive practices and health beliefs were generally favourable in this population, and this may have contributed to the relatively low malaria prevalence observed. Trust in health information and positive health beliefs were the factors most strongly associated with caregivers' willingness to vaccinate their children. Strengthening healthcare-worker engagement, improving vaccine communication, and addressing caregivers' informational gaps are recommended to support effective and equitable malaria vaccine uptake.

## Introduction

Malaria remains a leading cause of morbidity and mortality in Nigeria, particularly among children under five years and pregnant women. Nigeria accounts for the highest proportion of global malaria cases and deaths, with children under five bearing a disproportionate burden [1]. Despite ongoing control efforts, malaria transmission persists, especially in rural and semi-urban communities where environmental and socioeconomic conditions favour mosquito breeding.

Preventive practices such as the use of insecticide-treated nets (ITNs), environmental sanitation, and prompt healthcare-seeking behaviour are critical in reducing the malaria burden. However, adherence to these practices remains suboptimal due to limited awareness, cultural beliefs, poverty, and poor access to healthcare services [2]. In many communities, including those in the Akoko area of Ondo State, gaps persist between knowledge and actual practice of malaria prevention.

The introduction of malaria vaccines, particularly the RTS,S/AS01 and R21/Matrix-M vaccines, represents a major milestone in malaria control. These vaccines have been recommended by the World Health Organization as complementary interventions to existing preventive measures [1,3]. However, the success of these vaccines depends largely on caregiver acceptance, awareness, and trust in the health system.

Ondo State has recently strengthened its routine immunisation system through the rollout of the R21/Matrix-M malaria vaccine on the 2^nd^ of February, 2026, aimed at preventing childhood infections. This rollout provides an opportunity to assess caregiver attitudes toward the acceptability of vaccines, including malaria vaccines. Understanding how communities respond to new vaccination programmes is therefore crucial for effective implementation.

Emerging evidence from Nigeria suggests that while willingness to accept malaria vaccines may be relatively high, awareness remains low and vaccine hesitancy persists [4,5]. Hesitancy is often driven by concerns about safety, fear of side effects, misinformation, and lack of trust in government health programmes. Furthermore, socioeconomic factors such as education, income, and access to healthcare significantly influence both malaria preventive practices and vaccine acceptance.

Despite these findings, there is limited context-specific data from Southwest Nigeria, particularly from semi-rural settings such as Akoko. Additionally, most studies assess caregivers' willingness to vaccinate against malaria in isolation and do not examine its relationship with existing preventive practices or assess Health Belief Model constructs alongside it. There is also inadequate exploration of the socioeconomic and behavioural drivers of hesitancy. Addressing these gaps is essential for designing targeted and effective malaria control interventions.

Guided by the Health Belief Model (HBM) [6], this study assessed caregivers' willingness to vaccinate their children against malaria and identified the factors associated with this willingness among caregivers of children under five years in Akoko, Ondo State, Nigeria. The HBM is a widely used theoretical framework for understanding health-related decision-making and preventive behaviours [6], positing that individuals' engagement in health actions is influenced by their perceived susceptibility to a disease, perceived severity of its consequences, perceived benefits of an intervention, perceived barriers to action, cues to action, and self-efficacy. This study also provides baseline data on malaria preventive practices, malaria prevalence among children under five, and awareness of vaccine availability in this setting.

## Materials and methods

**Figure 1:**
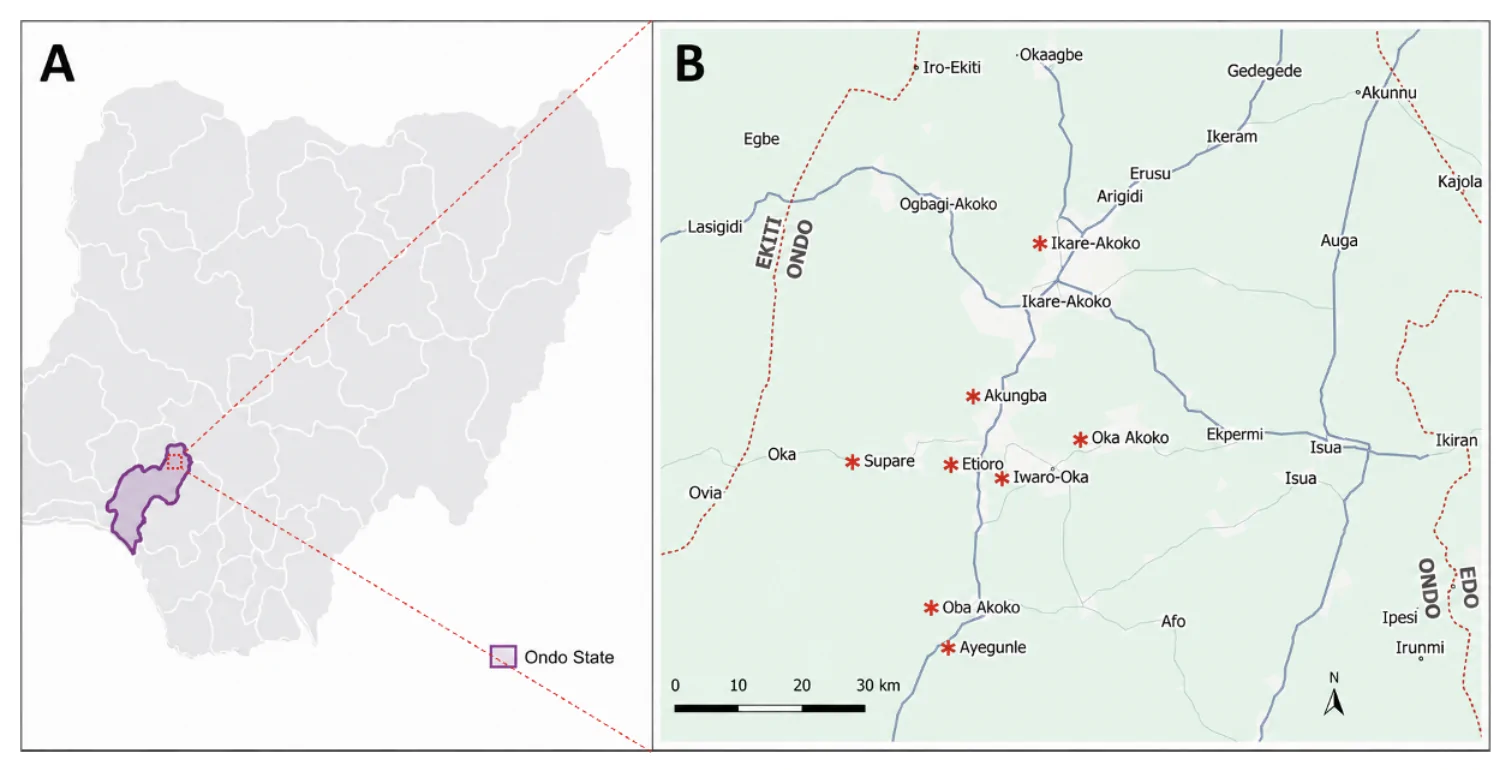
A: Map of Nigeria showing the location of Ondo State. B: Location of the eight sampled communities in the Akoko area (Oba, Ayegunle, Etioro, Akungba, Ikare, Oka, Iwaro and Supare).

The study was conducted in the Akoko area of Ondo State, Nigeria, which comprises a mix of rural and semi-urban communities predominantly situated in the rocky outcrop areas of northern Ondo State. The sampled communities included Oba (7.3759°N, 5.7234°E), Ayegunle (7.3455°N, 5.6943°E), Etioro (7.4380°N, 5.7239°E), Akungba (7.4740°N, 5.7379°E), Ikare (7.5248°N, 5.7669°E), Oka (7.4570°N, 5.8011°E), Iwaro (7.4433°N, 5.7644°E) and Supare (7.3812°N, 5.6248°E) (Figure 1). The predominant traditional occupations in these communities include large-scale agriculture, pastoralism and trading. The climate is classified as tropical wet-and-dry (savanna), with an average annual temperature of 24°C, annual rainfall of 1500 mm, and relative humidity of 75-95% [7-9].

### Study design

This was a community-based cross-sectional study comprising a mix of rural and semi-urban communities with varying levels of access to healthcare services. The sample size was determined using the Cochrane formula for cross-sectional studies, n = Z^2^pq / d^2^. Assuming a prevalence (p) of 50%, a 95% confidence level (Z = 1.96), and a margin of error (d) of 5%, a minimum sample size of 384 was calculated and adjusted upward for a 15% anticipated non-response rate.

A multistage sampling technique was used. In the first stage, two Local Government Areas (LGAs) Akoko Northeast and Akoko Southwest were purposively selected, as they are the predominantly urban LGAs in the area. Akoko Northeast was specifically included because it hosts the largest General Hospital in the area, and caregivers in this LGA were therefore expected to have higher vaccine awareness. No intermediate ward-selection stage was carried out; communities were selected directly within the two LGAs. In the second stage, eight communities with at least one primary health care center were randomly selected, seven from Akoko Southwest, and one from Akoko Northeast based on logistic accessibility. Within each selected community, a sampling frame of households was established with the assistance of local guides. A random starting point was determined, after which every third household was selected using systematic random sampling. In each selected household, all eligible caregivers of children under five years were identified and invited to participate. Inclusion criteria were caregivers of children under five years who had resided in the community for at least three months and who provided informed consent to participate.

### Data collection and quality control

Field data collection was conducted during a structured academic field practical exercise. A cohort of 100 undergraduate students from the Department of Public Health, Adekunle Ajasin University, participated in the field exercise. To ensure scientific rigour and minimise collection bias, all participating students underwent a standardised training session prior to fieldwork, focusing on the sampling protocol and data entry techniques. Field operations were directly supervised, and all field data were manually verified to maintain data integrity. Incomplete records, ambiguous entries, or unverified samples were excluded from the final analysis dataset.

Data were collected using a structured, interviewer-administered questionnaire designed to capture information relevant to the study objectives. The questionnaire was adapted, in part, from instruments used in previous studies on vaccine willingness with the Health Belief Model items developed by the author. The questionnaire was divided into seven sections: socio-demographic characteristics, malaria preventive practices, malaria prevalence, awareness, vaccine acceptability, Health Belief Model constructs, and socioeconomic characteristics. The Health Belief Model assessed six constructs: perceived severity (2 items), perceived susceptibility (1 item), perceived benefits (2 items), perceived barriers (3 items), cues to action (3 items), and self-efficacy (2 items). No formal pilot study was conducted prior to data collection.

### Malaria diagnosis

A finger-prick blood sample was collected from each eligible child using sterile lancets, and testing was conducted using WHO-approved malaria rapid diagnostic test (RDT) kits (Abbot Bioline Malaria Ag P. f, LOT: 05CDJ072A), with results read according to the manufacturer's instructions within the recommended time frame. Standard safety and infection-prevention protocols were followed throughout. Consent for the caregiver interview and consent for the child's RDT test were sought separately, and not all caregivers who completed the interview agreed to have their child tested; this accounts for the smaller sample size (n = 434) in the malaria prevalence analysis compared with the full caregiver sample (n = 498). Children who tested positive were referred to the nearest health facility for appropriate treatment. Demographic and clinical information for each child tested, including age, gender, and RDT result, was documented on a result form attached to each questionnaire [10].

### Data analysis

Data were entered into Microsoft Excel, cleaned, coded, and exported to R statistical software (version 4.x) for analysis. Statistical significance was set at p < 0.05. Socio-demographic and socioeconomic characteristics of caregivers were summarised using descriptive statistics [11]. Associations between categorical variables were assessed using the Pearson chi-square test.

Malaria preventive practices were assessed using eight items covering ownership and use of insecticide-treated nets (ITNs), environmental management, insecticide use, household screening, use of local herbs, and treatment-seeking behaviour. Each item was assigned a predefined score according to the response. Ownership of an ITN, use of insecticide spray, and use of window/door screens were scored 1 for 'Yes' and 0 for 'No'. Frequency of child ITN use, bush clearing, and removal of stagnant water were scored 0 for 'Never', 1 for 'Sometimes', and 2 for 'Always'. Use of local herbs for malaria prevention was reverse-scored, with 'No' assigned 1 and 'Yes' assigned 0. Appropriate treatment-seeking behaviour was assigned 1 and inappropriate treatment-seeking behaviour 0. Scores were summed to generate a composite practice score ranging from 0 to 11, with higher scores indicating more favourable malaria preventive practices. Scores of 0–5 were classified as poor practice, 6–8 as moderate practice, and 9–11 as good practice.

Health Belief Model (HBM) constructs were assessed using 13 questionnaire items. Responses were scored on a five-point Likert scale ranging from strongly disagree (1) to strongly agree (5). Negatively worded items assessing perceived barriers were reverse-coded so that higher scores consistently represented more favourable health beliefs towards malaria vaccination.

The items assessed six constructs: perceived severity (possible score range 2–10), perceived susceptibility (1–5), perceived benefits (2–10), perceived barriers (3–15), cues to action (3–15), and self-efficacy (2–10). Construct scores were calculated by summing the relevant item scores. An overall HBM score was obtained by summing all 13 items, giving a possible score range of 13–65. Participants were categorised as having low HBM (13–30), moderate HBM (31–48), or high HBM (49–65). Continuous HBM construct scores were summarised using means and standard deviations, while HBM categories were presented as frequencies and percentages.

Multivariable binary logistic regression was performed to identify factors independently associated with caregivers' willingness to vaccinate their children against malaria (the dependent variable). Continuous variables were entered as continuous predictors, and categorical variables as factors with pre-specified reference categories. Multicollinearity among independent variables was assessed using the Variance Inflation Factor (VIF). Adjusted odds ratios (AORs), 95% confidence intervals (CIs), and p-values were reported, with p < 0.05 considered statistically significant [12]. All variables were included in the multivariable logistic regression model regardless of their statistical significance in the bivariate analysis. Variables were excluded only where multicollinearity was indicated by the VIF.

### Ethical considerations

This study and its field practical protocols were reviewed and approved by the Human Research Ethics Committee, Research Management Office, Centre for Research and Development, Adekunle Ajasin University (Approval Number: 21032ER-C15). Informed consent was obtained from all participants prior to data collection.

## Results

### Study participants

A total of 498 caregivers of children under five years participated in the study. The mean (± SD) age of caregivers was 34.5 ± 11.0 years, and most were female (87.1%), married (88.8%), had secondary education (51.2%), and were traders (35.3%). A vast majority were Christians (81.9%), lived in large households (85.9%), had middle income level (50.4%), and had low asset levels (79.3%). Most lived in close proximity (less than 1 km) to at least one health facility (61.2%). Detailed socio-demographic and socioeconomic characteristics are presented in Supplementary Table S1. Of the 498 caregivers interviewed, 434 (87.1%) additionally consented to malaria RDT testing of their child; the malaria prevalence analysis (Table 2) is therefore based on this subset.

### Caregivers' malaria preventive practices

Most caregivers reported appropriate malaria preventive practices, particularly ownership of mosquito nets (72.5%), regular use of insecticide-treated nets by their children (44.6%), bush clearing around the home (72.5%), removal of stagnant water (71.1%), use of insecticide spray (63.5%), use of window screens (84.5%), and appropriate treatment-seeking behaviour (94.2%). However, the use of local herbal remedies (65.9%) and mosquito coils (32.5%) was still reported by a proportion of caregivers (Supplementary Table S2).

Preventive practice was significantly associated with caregivers' educational attainment (p = 0.029); caregivers with higher educational levels were more likely to demonstrate good malaria preventive practices than those with lower educational attainment. No significant associations were observed between preventive practice and age, gender, marital status, occupation, religion, household socioeconomic status, or access to healthcare (Table 1).

**Table 1:** Association (Pearson's Chi-squared test) between malaria preventive practice and socio-demographic and socioeconomic characteristics of caregivers of under-five children in Akoko, Ondo State.

Characteristic	Good (n=201) n (%)	Moderate (n=250) n (%)	Poor (n=47) n (%)	P value
**Age group (years)**				0.14
≤ 20	7 (3.5%)	6 (2.4%)	0 (0.0%)	
20–40	161 (80.0%)	186 (74.4%)	38 (80.9%)	
41–60	26 (13.0%)	54 (21.6%)	7 (14.8%)	
> 60	7 (3.5%)	4 (1.6%)	2 (4.3%)	
**Gender**				> 0.9
Female	175 (87.1%)	217 (86.8%)	42 (89.4%)	
Male	26 (12.9%)	33 (13.2%)	5 (10.6%)	
**Marital status**				0.3
Married	176 (87.5%)	224 (89.6%)	42 (89.3%)	
Single	16 (8.0%)	13 (5.2%)	2 (4.3%)	
Widowed	6 (3.0%)	5 (2.0%)	0 (0.0%)	
Divorced	3 (1.5%)	8 (3.2%)	3 (6.4%)	
**Education**				0.029
No formal education	4 (2.0%)	15 (6.0%)	2 (4.3%)	
Primary	21 (10.4%)	35 (14.0%)	5 (10.6%)	
Secondary	100 (49.8%)	123 (49.2%)	32 (68.1%)	
Tertiary	76 (37.8%)	77 (30.8%)	8 (17.0%)	
**Occupation**				0.6
Artisan	56 (27.9%)	85 (34.0%)	16 (34.0%)	
Civil servant	26 (12.9%)	30 (12.0%)	2 (4.3%)	
Farmer	21 (10.4%)	28 (11.2%)	5 (10.6%)	
Trader	77 (38.3%)	82 (32.8%)	17 (36.2%)	
Unemployed	13 (6.5%)	16 (6.4%)	6 (12.8%)	
Other	8 (4.0%)	9 (3.6%)	1 (2.1%)	
**Religion**				0.2
Christian	161 (80.1%)	208 (83.2%)	39 (82.9%)	
Islam	39 (19.4%)	38 (15.2%)	6 (12.8%)	
Traditional	1 (0.5%)	4 (1.6%)	2 (4.3%)	
**Household size**				0.2
Large	167 (83.1%)	218 (87.2%)	43 (91.5%)	
Moderate	34 (16.9%)	32 (12.8%)	4 (8.5%)	
**Housing type**				0.2
Brick	14 (7.0%)	31 (12.4%)	8 (17.0%)	
Cement	165 (82.1%)	192 (76.8%)	32 (68.1%)	
Mud	22 (10.9%)	27 (10.8%)	7 (14.9%)	
**Asset level**				> 0.9
Low	159 (79.1%)	200 (80.0%)	36 (76.6%)	
Moderate	24 (11.9%)	28 (11.2%)	6 (12.8%)	
High	18 (9.0%)	22 (8.8%)	5 (10.6%)	
**Income level**				0.6
Low	48 (23.9%)	70 (28.0%)	16 (34.0%)	
Middle	103 (51.2%)	125 (50.0%)	23 (49.0%)	
High	50 (24.9%)	55 (22.0%)	8 (17.0%)	
**Distance to nearest health facility**				0.5
Far	12 (6.0%)	15 (6.0%)	5 (10.6%)	
Near	189 (94.0%)	235 (94.0%)	42 (89.4%)	
**Transport Cost to Nearest Health Facility**				0.7
High Cost	42 (20.9%)	45 (18.0%)	8 (17.0%)	
Low Cost	159 (79.1%)	205 (82.0%)	39 (83.0%)	
**Location**				0.7
Akungba	44 (22.0%)	49 (19.6%)	7 (15.0%)	
Ayegunle	16 (8.0%)	26 (10.4%)	8 (17.0%)	
Etioro	19 (9.5%)	28 (11.2%)	3 (6.5%)	
Ikare	44 (22.0%)	49 (19.6%)	7 (15.0%)	
Iwaro	21 (10.0%)	24 (9.6%)	5 (10.5%)	
Oba	17 (9.0%)	28 (11.2%)	5 (10.5%)	
Oka	19 (9.5%)	27 (10.8%)	4 (8.5%)	
Supare	21 (10.0%)	19 (7.6%)	8 (17.0%)	

### Malaria prevalence among under-five children

Overall, 10.1% of under-five children tested had asymptomatic *Plasmodium falciparum* malaria during the survey. Malaria prevalence differed significantly across age groups (p = 0.008), with children aged 3-5 years experiencing a higher prevalence than younger children. No significant difference in malaria prevalence was observed by gender (Table 2).

**Table 2:** Prevalence of malaria among under-five children by age and gender in Akoko, Ondo State.

Age/gender category	Negative (n=390) n (%)	Positive (n=44) n (%)	P value
**Age (years)**			0.008
< 1	4 (1.0%)	3 (6.8%)	
1-2	97 (24.9%)	7 (15.9%)	
3-5	289 (74.1%)	34 (77.3%)	
**Gender**			0.16
Female	214 (54.9%)	29 (65.9%)	
Male	176 (45.1%)	15 (34.1%)	

### Awareness and acceptability of the malaria vaccine

Most caregivers had heard of the malaria vaccine (77.9%), primarily through the health system (61.4%), and the majority reported trusting the information they received (75.3%). Overall, willingness to vaccinate was high, with most caregivers indicating willingness to vaccinate their children (87.9%) and recommending the vaccine to others (86.5%). Mothers (40.6%) were the predominant decision-makers regarding childhood vaccination (Table 3).

**Table 3:** Awareness and acceptability of the malaria vaccine among caregivers of under-five children in Akoko, Ondo State.

Characteristic	n (%)
**Heard of malaria vaccine**	
Yes	388 (77.9%)
No	110 (22.1%)
**Source of information**	
Community/education	29 (5.8%)
Health system	298 (59.9%)
Mass media	61 (12.2%)
No information	110 (22.1%)
**Trust the information**	
Yes	375 (75.3%)
No	123 (24.7%)
**Willingness to vaccinate child**	
Yes	438 (87.9%)
No	60 (12.1%)
**Recommend to others**	
Yes	431 (86.5%)
No	67 (13.5%)
**Decision maker**	
Father	111 (22.3%)
Mother	202 (40.6%)
Both	159 (31.9%)
Grandparent	26 (5.2%)

**Figure 2:**
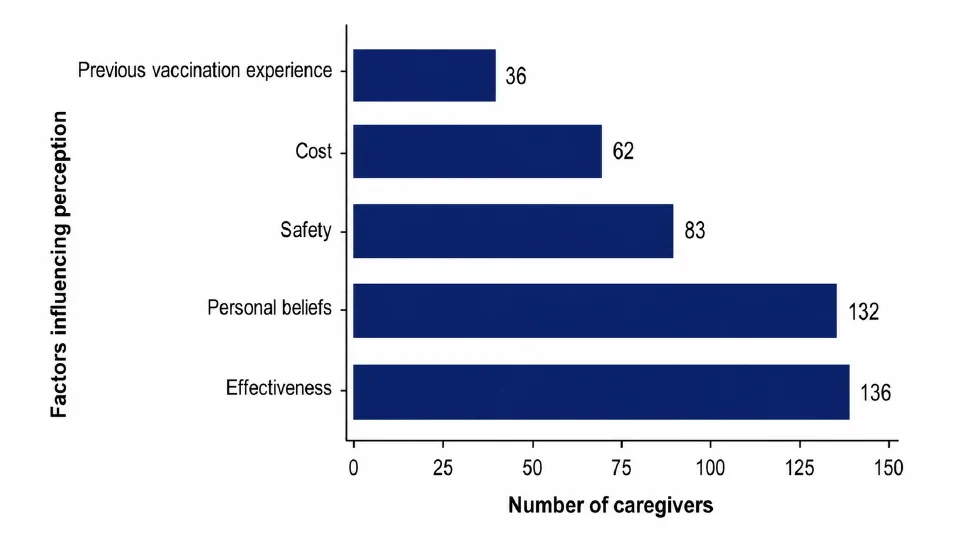
Factors influencing caregivers' perceptions of the malaria vaccine in Akoko, Ondo State.

The most frequently reported factors influencing caregivers' perceptions of the malaria vaccine were vaccine effectiveness (32.5%), personal beliefs (26.5%), safety (16.7%), cost (12.4%), and previous vaccination experiences (7.2%) Caregivers could select more than one factor influencing their perception of the malaria vaccine (Figure 2).

### Health Belief Model constructs

Caregivers demonstrated generally favourable health beliefs towards malaria vaccination. Mean scores for perceived severity, perceived susceptibility, perceived benefits, perceived barriers, cues to action, self-efficacy, and the overall HBM score are presented in Table 4. Most caregivers were classified as having a high overall HBM score (59.6%).

**Table 4:** Health Belief Model constructs among caregivers of under-five children in Akoko, Ondo State (n=498).

Characteristic	Value (mean ± SD)
**HBM construct**	
Severity	8.5 ± 2.4
Susceptibility	3.9 ± 1.2
Benefit	8.4 ± 2.3
Barrier	7.0 ± 2.6
Action cues	12.5 ± 3.2
Efficacy	8.0 ± 2.2
Total	48.3 ± 13.9
**Overall HBM category, n (%)**	
Low	40 (8.1%)
Moderate	161 (32.3%)
High	297 (59.6%)

### Factors associated with willingness to vaccinate

Religion, trust in health information, and HBM category were significantly associated with willingness to vaccinate (p = 0.014, p = 0.002 and p < 0.001, respectively). Caregivers who were Christian (83.8%), who trusted the information provided by the health system (77.6%), and who had high positive beliefs (64.0%) were more willing to vaccinate their children. Other sociodemographic, socioeconomic, healthcare-access, malaria-preventive, and vaccine-awareness factors were not statistically significant (Table 5).

**Table 5:** Bivariate analysis (Pearson's Chi-squared test) of factors associated with malaria vaccine acceptability among caregivers of under-five children in Akoko, Ondo State.

Characteristic	No (n =60) n (%)	Yes (n=438) n (%)	P value
**Age group (years)**			0.3
< 20	2 (3.3%)	11 (2.5%)	
20–40	51 (85.1%)	334 (76.3%)	
41–60	5 (8.3%)	82 (18.7%)	
> 60	2 (3.3%)	11 (2.5%)	
**Gender**			>0.9
Female	52 (86.7%)	382 (87.2%)	
Male	8 (13.3%)	56 (12.8%)	
**Marital status**			0.6
Single	4 (6.7%)	27 (6.2%)	
Married	55 (91.7%)	387 (88.3%)	
Widowed	0 (0%)	11 (2.5%)	
Divorced	1 (1.6%)	13 (3.0%)	
**Education**			0.3
No formal education	3 (5.0%)	18 (4.1%)	
Primary	3 (5.0%)	58 (13.2%)	
Secondary	35 (58.3%)	220 (50.2%)	
Tertiary	19 (31.7%)	142 (32.5%)	
**Occupation**			0.7
Artisan	16 (26.7%)	141 (32.2%)	
Civil servant	10 (16.7%)	48 (10.9%)	
Farmer	7 (11.7%)	47 (10.7%)	
Trader	22 (36.7%)	154 (35.2%)	
Unemployed	4 (6.6%)	31 (7.1%)	
Other	1 (1.6%)	17 (3.9%)	
**Religion**			0.014
Christian	41 (68.3%)	367 (83.8%)	
Islam	17 (28.3%)	66 (15.1%)	
Traditional	2 (3.3%)	5 (1.1%)	
**Household size**			0.7
Large	53 (88.3%)	375 (85.6%)	
Moderate	7 (11.7%)	63 (14.4%)	
**Income level**			0.3
Low	21 (35.0%)	113 (25.8%)	
Middle	25 (41.7%)	226 (51.6%)	
High	14 (23.3%)	99 (22.6%)	
**Asset Level**			0.8
Low	49 (81.6%)	346 (79.0%)	
Moderate	7 (11.7%)	51 (11.6%)	
High	4 (6.7%)	41 (9.4%)	
**Location**			
Akungba	8 (13.3%)	92 (21.0%)	
Ayegunle	10 (16.7%)	40 (9.1%)	
Etioro	8 (13.3%)	42 (9.6%)	
Ikare	14 (23.3%)	86 (19.6%)	
Iwaro	8 (13.3%)	42 (9.6%)	
Oba	7 (11.7%)	43 (9.8%)	
Oka	3 (5.1%)	47 (10.7%)	
Supare	2 (3.3%)	46 (10.6%)	
**Distance to nearest health facility**			0.088
Far	7 (11.7%)	25 (5.7%)	
Near	53 (88.3%)	413 (94.3%)	
**Transport cost to nearest health facility**			0.12
High	16 (26.7%)	79 (18.0%)	
Low	44 (73.3%)	359 (82.0%)	
**Malaria preventive practice level**			0.6
Good	23 (38.3%)	178 (40.6%)	
Moderate	29 (48.3%)	221 (50.5%)	
Poor	8 (13.4%)	39 (8.9%)	
**Heard of malaria vaccine**			0.14
No	18 (30.0%)	92 (21.0%)	
Yes	42 (70.0%)	346 (79.0%)	
**Trust the information**			0.001
No	25 (41.7%)	98 (22.4%)	
Yes	35 (58.3%)	340 (77.6%)	
**Health Belief Model level**			<0.001
Low	3 (5.0%)	37 (8.4%)	
Moderate	40 (66.7%)	121 (27.6%)	
High	17 (28.3%)	280 (64.0%)	

### Multivariable analysis of willingness to vaccinate

After adjustment for potential confounders in the multivariable logistic regression model, three variables remained independently associated with willingness to vaccinate (Table 6). Caregivers who did not trust the information they received about the malaria vaccine had significantly lower odds of being willing to vaccinate than those who trusted the information (AOR = 0.16; 95% CI: 0.05-0.53; p = 0.002). Likewise, caregivers who reported that previous vaccination experiences influenced their vaccination decision had significantly lower odds of willingness to vaccinate (AOR = 0.24; 95% CI: 0.09-0.71; p = 0.009).

**Table 6:** Multivariable logistic regression analysis of predictors of malaria vaccine acceptability among caregivers of under-five children in Akoko, Ondo State (AOR = Adjusted Odds Ratio, CI = Confidence Interval).

Characteristic	AOR	95% CI	P value
Age	1.00	0.97, 1.04	0.9
**Gender (ref: female)**			
Male	0.82	0.32, 2.27	0.7
**Marital Status (ref: married)**			
Single	0.75	0.23, 2.99	0.7
Divorced	3.15	0.34, 93.0	0.4
**Education (ref: tertiary)**			
No Formal Education	0.87	0.16, 7.79	0.9
Primary	2.73	0.72, 14.0	0.2
Secondary	0.62	0.29, 1.31	0.2
**Occupation (ref: civil servant)**			
Artisan	1.46	0.49, 4.25	0.5
Farmer	0.86	0.24, 3.30	0.8
Trader	1.41	0.51, 3.81	0.5
Unemployed	1.19	0.30, 5.42	0.8
**Religion (ref: Christianity)**			
Islam	0.48	0.23, 1.02	0.05
Traditional	0.14	0.02, 1.62	0.078
**Household Size**	0.96	0.83, 1.13	0.6
**Number of under-fives (ref: 1)**			
2	0.96	0.47, 2.03	>0.9
3	1.58	0.47, 6.57	0.5
**Distance to nearest health facility (ref: Near)**			
Far	0.81	0.37, 1.81	0.6
**Time to nearest health facility (ref: Near)**			
Far	0.66	0.21, 2.24	0.5
**Transport cost (ref: low cost)**			
High Cost	0.72	0.27, 1.95	0.5
**Treatment seeking practice (ref: Appropriate)**			
Inappropriate	0.71	0.23, 2.66	0.6
**Child had malaria in last 3 months (ref: Yes)**			
No	1.00	0.53, 1.88	>0.9
**Heard of malaria vaccine (ref: Yes)**			
No	7.36	0.94, 163	0.1
**Source of information (ref: health system)**			
Community/education	0.31	0.09, 1.11	0.062
Mass media	0.54	0.21, 1.48	0.2
No information	0.28	0.01, 2.79	0.3
**Trust the information (ref: Yes)**			
No	0.16	0.05, 0.53	0.002
**Cost influence on acceptability (ref: No)**			
Yes	2.54	0.86, 9.31	0.12
**Personal beliefs influence (ref: No)**			
Yes	0.78	0.35, 1.78	0.6
**Vaccine effectiveness influence (ref: No)**			
Yes	2.06	0.85, 5.41	0.12
**Vaccine safety influence (ref: No)**			
Yes	0.74	0.31, 1.91	0.5
**Previous vaccination experience influence (ref: No)**			
Yes	0.24	0.09, 0.71	0.009
**Health Belief Model Score**	1.04	1.00, 1.08	0.03
**Asset level (ref: high)**			
Low	0.88	0.24, 2.61	0.8
Moderate	1.19	0.26, 5.14	0.8
**Income level (ref: high)**			
Low	0.83	0.34, 1.98	0.7
Middle	1.99	0.85, 4.60	0.11

In contrast, higher overall HBM scores were positively associated with willingness to vaccinate; each one-point increase in the HBM score was associated with approximately 4% higher odds of reported willingness to vaccinate against malaria (AOR = 1.04; 95% CI: 1.00-1.08; p = 0.030).

## Discussion

This study describes the malaria preventive practices, Health Belief Model constructs, and factors associated with willingness to vaccinate among caregivers in the Akoko area of Ondo State. About 90% of caregivers had moderate-to-good malaria preventive practices, suggesting good knowledge of malaria transmission, prevention and control in the area. This is in line with previous reports from other parts of the country [13,14]. Malaria prevention practices were predominantly the use of long-lasting insecticide-treated nets, window screens, and environmental sanitation, similar to reports from other parts of Nigeria and West Africa [13,15-17], but differing from the reports of Adeleke *et al.* [18] and Edefo and Bawuah [19].

Education was significantly associated with malaria preventive practices; caregivers with tertiary education were more frequently associated with good preventive practices. This agrees with the reports of Adeleke *et al.* [18], Koroma *et al.* [16], Edefo and Bawuah [19], and Akinrinade *et al.* [20], and suggests that education plays an important role in promoting appropriate malaria prevention behaviours. Caregivers with higher educational attainment may have better access to health information, greater awareness of recommended malaria prevention strategies, and be better equipped economically to practise consistent preventive measures, all of which may contribute to improved malaria preventive practices [19,21].

The prevalence of asymptomatic *P. falciparum* infection in this study was 10.1%, lower than previously reported by Balogun *et al.* [22] and Oluwafemi *et al.* [23] in the same region. Several factors could plausibly contribute to this difference, including preventive practices, the recent vaccine rollout, and differences in study population or timing, but the cross-sectional design does not allow their relative contributions to be resolved. This lower prevalence could reflect the good malaria preventive practices observed in this population, or the recent introduction of the malaria vaccine; many caregivers also reported that their children had received at least one dose of the malaria vaccine. Malaria prevalence varied significantly with age, with a higher percentage of infected children aged 3-5 years, this age-related pattern may reflect a combination of factors, such as differences in exposure and the gradual acquisition of partial immunity with cumulative exposure to infection [24].

There was high awareness of the malaria vaccine in this population, with most caregivers obtaining information from community healthcare workers, hospitals and clinics. Most caregivers trusted this information and were willing to vaccinate their children. Reports on vaccine awareness in Nigeria and Africa vary, with some studies reporting high awareness similar to this study [15,16,25] and others observing low awareness [4,14,26-28]. This variation may reflect regional differences in the uptake and deployment of vaccination programmes. Willingness to vaccinate in this study was, however, lower than the pooled estimate of 95.3% reported by Sulaiman *et al.* [29] for low- and middle-income countries, who measured caregivers' self-reported willingness to accept the RTS, S malaria vaccine.

The decision to vaccinate was mostly made by mothers or both parents jointly, which is expected as mothers typically accompany children for vaccination and receive health information directly from healthcare providers. Several studies have reported a link between female or biological-parent caregiving and higher vaccine uptake [15,30-32].

The factors that most shaped caregivers' perceptions of the malaria vaccine were vaccine effectiveness, personal beliefs, and safety, consistent with previous studies [4,15,26,27,29]. Perceptions of effectiveness may be shaped by past experience with childhood vaccination programmes such as polio and measles [33]. Personal beliefs about vaccination have similarly been linked to perceptions of disease seriousness and confidence that vaccines are life-saving with few side effects [34]. Sulaiman *et al.* [29] also reported that previous vaccine-related adverse effects are a significant barrier to completing childhood immunisation, and Dube *et al.* [35] suggested that childhood beliefs about vaccination can shape vaccination acceptance in adulthood.

Religion, trust in vaccine information, and health beliefs were significantly associated with vaccine acceptance in the bivariate analysis. Christian caregivers were more willing to accept the vaccine than caregivers of other religions, a pattern also observed during the polio vaccination programme, where some Muslim communities believed the vaccine was unsafe and could cause infertility, HIV, or cancer [36]. Religious leaders have been reported to shape community perceptions and vaccination decisions [26,27,33], and engaging them has been shown to effectively address misconceptions and improve vaccine acceptance in Nigeria [37].

Most caregivers who trusted vaccine information were willing to vaccinate their children. Caregivers have also been reported to be willing to vaccinate in areas of comparatively low awareness [4,14-16,25-27], possibly reflecting the burden and severity of malaria in endemic regions and a generally high level of trust in healthcare workers. A similar trend was reported in Nigeria and Cameroon by Ibrahim Adamu *et al.* [26], Aboki *et al.* [30], and Abiache *et al.* [32], who found that caregivers obtaining information from community health events and healthcare providers were more likely to accept the vaccine than those relying on family, friends, or social media. This underscores the role of healthcare providers in the success of health intervention programmes, and the need to invest in capacity-building so that health workers can provide accurate information and counter misinformation [30,38]. It also highlights the importance of both the source and content of health communication messages; culturally appropriate messaging delivered through the right channels is essential for correct information uptake [14,30].

The positive association between HBM score and willingness to vaccinate indicates that caregivers with more favourable health beliefs were more willing to vaccinate their children. High scores for perceived severity, perceived benefits, cues to action, and self-efficacy suggest that caregivers recognised the seriousness of malaria and the value of vaccination while expressing confidence in their ability to have their children vaccinated. This is consistent with previous reports that positive vaccine perception significantly influences willingness to vaccinate [27,34]. In contrast, Idang *et al.* [28] reported lower positivity in Cameroon, illustrating a gap between general support for vaccination and actual willingness to vaccinate in some settings. This highlights the importance of accurate, comprehensive caregiver education through health campaigns, community engagement, and targeted communication [36].

Trust in malaria vaccine information, previous vaccination experiences, and the overall HBM score were identified as independently associated with willingness to vaccinate. Trust emerged as one of the strongest correlates: caregivers who did not trust the information they received were considerably less likely to be willing to vaccinate than those who trusted it, underscoring the critical role of trust in health communication [30]. Other African studies have similarly reported higher vaccine uptake among caregivers who relied on health workers to verify information [28,30,32]; Ndoula *et al.* [38] found that caregivers who trusted health workers were six times more likely to vaccinate their children. Trust is fundamental to immunisation programme success because it shapes perceptions of vaccine safety, effectiveness, and the credibility of health authorities [32]. Strengthening risk communication through trusted sources such as healthcare workers, community leaders, and government health programmes may therefore improve confidence in, and uptake of, malaria vaccination [38].

Previous vaccination experiences were also significantly associated with willingness to vaccinate: caregivers who reported that such experiences influenced their decision had lower odds of being willing to vaccinate against malaria than those who did not. Although, the study did not distinguish between the specific nature of these experiences, the finding suggests that caregivers' prior interactions with childhood immunisation services may be associated with subsequent vaccination decisions. Evidence from the COVID-19 pandemic similarly shows that vaccine attitudes and trust established during one immunisation experience continue to shape subsequent vaccination behaviours [39]. This underscores the need to ensure positive caregiver experiences throughout routine immunisation services, including effective communication, respectful care, and prompt management of vaccine-safety concerns.

The overall HBM score was positively associated with willingness to vaccinate, with each one-point increase raising the odds of willingness by approximately 4%. The mean HBM score of 48.3 out of a possible 60 suggests that caregivers generally perceived malaria as a serious disease, recognised their children's susceptibility, appreciated the benefits of vaccination, perceived relatively few barriers, responded to cues promoting vaccination, and expressed confidence in their ability to vaccinate their children. This supports the HBM as a useful framework for understanding malaria vaccine acceptance and suggests that interventions strengthening positive health beliefs may improve uptake [27]. Educational interventions emphasising the severity of malaria, vaccine effectiveness and safety, and addressing misconceptions and perceived barriers may therefore improve acceptance of malaria vaccines among caregivers [27].

This study has several limitations. First, the cross-sectional design does not permit causal inference, and observed associations should be interpreted accordingly. Second, participants were recruited from eight communities across two purposively selected LGAs, with an uneven distribution reflecting logistical accessibility. This, together with within-community household sampling, may have introduced clustering effects and selection bias, thus findings should be generalised beyond Akoko South-West with caution. Third, the questionnaire which was adapted in part from previous studies with the Health Belief Model items developed by the author and was not formally piloted or subjected to reliability assessment before deployment. Fourth, this study did not systematically capture whether children had already received a malaria vaccine dose. Finally, willingness to vaccinate was self-reported and not validated against actual uptake. Nonetheless, this study provides valuable, context-specific insights into the factors associated with caregivers' willingness to vaccinate their children against malaria in the Akoko area of Ondo State, relevant to future vaccination programmes.

## Conclusions

This study found generally good malaria preventive practices and favourable health beliefs among caregivers of under-five children in Akoko, Ondo State, which may partly explain the relatively low asymptomatic malaria prevalence observed. Trust in vaccine information and positive Health Belief Model scores were the factors most strongly associated with both malaria preventive practice and vaccine acceptability, while caregivers whose past vaccination experiences influenced their decision-making showed lower acceptance. These findings suggest that strengthening healthcare-worker engagement, improving the clarity and reach of vaccine communication, and directly addressing caregivers' informational gaps and misconceptions should be prioritised by policymakers and public health stakeholders designing malaria vaccine roll-out strategies, in order to support equitable and sustained vaccine uptake.

## Appendix

**Supplementary Table S1:** Socio-demographic and socioeconomic characteristics of caregivers of under-five children in Akoko, Ondo State.

Characteristic	n (%) / Mean ± SD
Age (years)	34.5 ± 11.0
**Age group**	
≤ 20	13 (2.6%)
20–40	385 (77.3%)
41–60	87 (17.5%)
> 60	13 (2.6%)
**Gender**	
Female	434 (87.1%)
Male	64 (12.9%)
**Marital status**	
Married	442 (88.8%)
Single	31 (6.2%)
Divorced	14 (2.8%)
Widowed	11 (2.2%)
**Education**	
No formal education	21 (4.2%)
Primary	61 (12.3%)
Secondary	255 (51.2%)
Tertiary	161 (32.3%)
**Occupation**	
Artisan	157 (31.6%)
Civil servant	58 (11.7%)
Farmer	54 (10.8%)
Trader	176 (35.3%)
Unemployed	35 (7.0%)
Other	18 (3.6%)
**Religion**	
Christian	408 (81.9%)
Islam	83 (16.7%)
Traditional	7 (1.4%)
**Household size**	
Large	428 (85.9%)
Moderate	70 (14.1%)
**Distance to nearest health facility**	
< 1km	305 (61.2%)
1-3km	163 (32.7%)
4-5km	30 (6.1%)
**Housing type**	
Brick	53 (10.6%)
Cement	389 (78.1%)
Mud	56 (11.3%)
**Asset level**	
High	45 (9.1%)
Moderate	58 (11.6%)
Low	395 (79.3%)
**Income level**	
High	113 (22.7%)
Middle	251 (50.4%)
Low	134 (26.9%)
**Location**	
Akungba	100 (20.0%)
Ayegunle	50 (10.1%)
Etioro	50 (10.1%)
Ikare	100 (20.0%)
Iwaro	50 (10.1%)
Oba	50 (10.1%)
Oka	50 (10.1%)
Supare	48 (9.5%)

**Supplementary Table S2:** Frequency distribution of malaria preventive practices among caregivers of under-five children in Akoko, Ondo State.

Characteristic	n (%)
**Own mosquito net**	
Yes	361 (72.5%)
No	137 (27.5%)
**Child sleeps under net**	
Always	222 (44.6%)
Sometimes	89 (17.8%)
Never	187 (37.6%)
**Bush clearing**	
Always	361 (72.5%)
Sometimes	110 (22.1%)
Never	27 (5.4%)
**Remove stagnant water**	
Always	354 (71.1%)
Sometimes	87 (17.5%)
Never	57 (11.4%)
**Use insecticide spray**	
Yes	316 (63.5%)
No	182 (36.5%)
**Use mosquito coils**	
Always	162 (32.5%)
Sometimes	127 (25.5%)
Never	209 (42.0%)
**Use window screen**	
Yes	416 (84.5%)
No	82 (16.5%)
**Use local herb**	
Yes	328 (65.9%)
No	170 (34.1%)
**Treatment seeking practice**	
Appropriate	469 (94.2%)
Inappropriate	29 (5.8%)
**Overall malaria preventive practice**	
**(mean ± SD = 8.72 ± 2.37)**	
Good	201 (40.4%)
Moderate	250 (50.2%)
Poor	47 (9.4%)
